# Development of Immunochromatographic Assay for the Rapid Detection of *Mycoplasma capricolum* subsp. *capripneumoniae* Antibodies

**DOI:** 10.3389/fmicb.2021.743980

**Published:** 2022-01-11

**Authors:** Zhen Zhu, Guanggang Qu, Changjiang Wang, Lei Wang, Jige Du, Qianlin Li, Zhiqiang Shen, Xiaoyun Chen

**Affiliations:** ^1^China Institute of Veterinary Drug Control, Beijing, China; ^2^Shandong Binzhou Animal Science and Veterinary Medicine Academy, Binzhou, China

**Keywords:** *Mycoplasma capricolum* subsp. *capripneumoniae*, recombinant P20 protein, colloidal gold-based immunochromatographic strip, serum antibody, rapid on-site diagnosis

## Abstract

*Mycoplasma capricolum* subsp. *capripneumoniae* (Mccp) is the cause of contagious caprine pleuropneumonia (CCPP), which is a highly significant respiratory disease in goats leading to significant economic losses in Africa and Asia. Currently available procedures for the diagnosis of CCPP have some limitations in sensitivity, specificity, operation time, requirement of sophisticated equipment or skilled personnel, and cost. In this study, we developed a rapid, sensitive, and specific colloidal gold-based immunochromatographic assay (GICA) strip for the efficient on-site detection of antibodies against Mccp in the serum within 10 min. For the preparation of this colloidal GICA strip, recombinant P20 protein, the membrane protein of Mccp, was expressed by *Escherichia coli* prokaryotic expression system after purification was used as the binding antigen in the test. The rabbit anti-goat immunoglobulin G labeled with the colloidal gold was used as the detection probe, whereas the goat anti-rabbit immunoglobulin G was coated on the nitrocellulose membrane as the control line. The concentration of the coating antibody was optimized, and the effectiveness of this colloidal GICA strip was evaluated. Our results proved that the detection limit of the test strip was up to 1:64 dilutions for the Mccp antibody-positive serum samples with no cross-reactivity with other pathogens commonly infecting small ruminants,including goat pox virus, peste des petits ruminants virus, foot-and-mouth disease virus type A, or other mycoplasmas. Moreover, the colloidal GICA strip was more sensitive and specific than the indirect hemagglutination assay for the detection of Mccp antibodies. The 106 clinical serum samples were detected by the colloidal GICA strip compared with the complement fixation test, demonstrating an 87.74% concordance with the complement fixation test. This novel colloidal GICA strip would be an effective tool for the cost-effective and rapid diagnosis of CCPP in the field.

## Introduction

Contagious caprine pleuropneumonia (CCPP) is a severe respiratory disease with high morbidity and mortality in goats (Ahaduzzaman, 2021). Its prevalence is considerably high and has a serious impact on goat farming throughout the globe, especially in Africa, the Middle East, and some parts of Asia. The economic losses caused by CCPP are estimated to be approximately US$507 million per annum (Yatoo et al., 2019). The disease is highly infectious and characterized by coughing, nasal discharge, and acute respiratory distress with extensive lesions in the lung (pneumonia) and pleura (Lorenzon et al., 2008; Yatoo et al., 2019). CCPP has been included in the list B of communicable diseases in animals by the World Organization for animal health^1^ because of its high contagiousness and serious impact on the socio-economy.

*Mycoplasma capricolum* subsp. *capripneumoniae* (Mccp), formerly referred to as *Mycoplasma* strain F38-type (Manso-Silván and Thiaucourt, 2019), is the causative pathogen of CCPP (Li et al., 2020) and was first isolated in Kenya. It belongs to the *Mycoplasma mycoides* cluster, including five closely related species, subspecies, or biotypes (Woubit et al., 2004). Mccp and the other pathogens in the *M. mycoides* cluster or peste des petits ruminants virus (PPRV) or pasteurellosis may induce similar respiratory symptoms in goats (Jean de Dieu et al., 2019), so that Mccp infection cannot usually be diagnosed by physical examination alone (Chota et al., 2019). In addition, challenges also exist in the culture isolation of the etiologic agent because of its specific medium requirements (Teshome and Sori, 2021). As a consequence, the diagnosis of the disease may be difficult in clinical practice. This may be the reason why CCPP was described as early as 1,873 in Algeria (Yatoo et al., 2019), but the description of its causative agent was delayed nearly 100 years (McMartin et al., 1980).

Although several methods have been conducted to the determination of CCPP (Woubit et al., 2004; Lorenzon et al., 2008; Tharwat and Al-Sobayil, 2017; Lin et al., 2018; Jean de Dieu et al., 2019), they have certain drawbacks in either ease-of-use, sensitivity, specificity, cost, or dependence on specialized equipment or expertise. Immunochromatographic assay (ICA) is a powerful technique based on immunochromatographic procedure, which has been frequently used for the rapid detection of various biological specimens, especially specific antigens and antibodies of many diseases (Liu et al., 2021). Compared with other laboratory-based diagnostics, ICA is more user-friendly, relatively inexpensive, and device-independent, which makes it feasible for field diagnosis of many diseases in a short time (Shyu et al., 2002). Prompt diagnosis is crucial for the effective control and monitoring of CCPP. This study aims to develop a cost-effective, specific, and sensitive colloidal gold-based immunochromatographic assay (GICA) strip, which can be used as an alternative assay for the rapid sero-surveillance and the on-site detection of antibodies against CCPP in goats.

## Materials and Methods

### Materials and Reagents

Recombinant *Escherichia coli* strain BL21 (DE3) expressing Mccp-P20 protein (*E. coli*-Mccp-P20) was constructed and provided by the China Institute of Veterinary Drug Control. The Mccp standard-positive serum [indirect hemagglutination assay (IHA) titer 1:8], Mccp standard-negative serum, serum samples for specificity test including *Mycoplasma ovipneumoniae* (Mo)-positive serum, goat pox virus (GTPV)-positive serum, PPRV-positive serum, foot-and-mouth disease virus type A (FMDV-A)-positive serum, *Mycoplasma mycoides* subsp. *capri* (Mmc)-positive serum, *Mycoplasma capricolum* subsp. *capricolum* (Mcc)-positive serum, and *E. coli* BL21 (DE3)-positive serum were prepared by the China Institute of Veterinary Drug Control. Kanamycin and isopropyl-β-D-thiogalactoside were obtained from Solarbio (Beijing, China). Hydrogen tetra-chloroaurate hydrate, trisodium citrate, potassium carbonate, and albumin from bovine serum were purchased from Sinopharm (Shanghai, China). The HisTrap FF column was obtained from Bestchrom (Shanghai) Bioscience Co., Ltd. (Shanghai, China). The rabbit anti-goat immunoglobulin G (IgG) and goat anti-rabbit IgG were obtained from Luoyang Bai Aotong Experimental Materials Center. Nitrocellulose (NC) membrane and glass cellulose membrane were products of Bioadvantage Co., Ltd. IHA antigen of CCPP was prepared by Lanzhou Veterinary Research Institute, Chinese Academy of Agricultural Science (Lanzhou, China).

### Expression, Purification, and Identification of the Recombinant P20 Protein

To express the recombinant P20 (rP20) protein, the MCCP P20 gene (sequence shown in the Supplementary Data) was subcloned from the pUC57-P20 vector into the pET28a(+) vector (EMD Millipore, San Diego, CA, United States), transformed into *E. coli* BL21 (DE3) (Novagen, Madison, WI, United States) under kanamycin (100 μg/mL) selection, and recombinant *E. coli* BL21 (DE3) expressing rP20 protein was constructed and named *E. coli*-Mccp-P20. The bacteria *E. coli*-Mccp-P20 was cultured overnight in Luria-Bertani media supplemented with 100 μg/mL kanamycin at 37°C with shaking at 180 rpm and transferred into a fresh medium until the optical density at a wavelength of 600 nm reached 0.6–0.8 and induced in the presence of 1 mmol/L isopropyl-β-D-thiogalactoside. To optimize the expression condition, the bacteria were induced at 15°C for 16 h and 37°C for 4 h. Bacterial cells in each group were collected and lysed, and the sediment of cell lysates was collected by centrifugation and dissolved in phosphate-buffered saline (PBS) (pH 7.2) with 8 mol/L urea, which was then loaded onto a HisTrap FF column. The purification was performed according to the manufacturer’s instructions. Finally, the purified and denatured recombinant protein was refolded by dialysis with a gradient of decreasing concentration of urea, and the refolded protein was stored at −80°C for further use.

Protein samples were separated by 12% sodium dodecyl sulfate–polyacrylamide gel electrophoresis and analyzed by Western blotting. Briefly, proteins were transferred by electroblotting onto a polyvinylidene fluoride membrane that was then blocked with 5% skimmed milk, followed by incubating with anti-His rabbit monoclonal antibody (1:2,000) for 1 h at room temperature. After washing in PBST (0.05% Tween 20 with 0.01 mol/L, PBS, pH 7.2), the membrane was incubated with horseradish peroxidase-conjugated goat anti-rabbit antibody (1:10,000) for 1 h at room temperature with gentle shaking. Finally, the blots were visualized using Chemistar ECL Western blotting detection system according to the manufacturer’s instructions.

### Preparation of Colloidal Gold Suspension

Colloidal gold with a diameter range of 20–40 nm was prepared according to the method reported previously (Yu et al., 2018). In brief, to get the most suitable size of colloidal gold particles, different volume ratios (1:0.5, 1:1, 1:1.6, and 1:2.5) of 10% trisodium citrate solution were dropped into the boiled solution under continuous stirring, and then the reaction mixture was boiled for another 10 min. The colloidal gold solution was cooled down to room temperature and then characterized on an ultraviolet spectrophotometer. The diameter of colloidal gold was calculated according to the following linear regression equation, λ = 0.4271X + 514.56 (λ, maximum absorption wavelength; X, particle diameter). It was finally stored at 4°C in the dark.

### Preparation of the Colloidal Gold-Labeled Rabbit Anti-goat Immunoglobulin G

To determine the optimal amount of the rabbit anti-goat IgG for conjugation with the colloidal gold solution, the rabbit anti-goat IgG was diluted to different concentrations (0, 1.2, 1.6, 2, 2.4, 2.8, and 3 μg/ml), and 5 μl of each was added into 1 ml of colloidal gold solution (pH 8.0) with slowly shaking for 5 min, followed by adding 0.1 ml of 10% sodium chloride solution into each tube with stirring for 5 min. With the increase of the IgG amount, the color of the mixtures changed from blue to red after incubation for 30 min at room temperature. The minimum concentration of antibody that made the red color of the mixture unchanged was regarded as the optimum concentration of rabbit anti-goat IgG for colloidal gold labeling. The rabbit anti-goat IgG-coated colloidal gold probe was prepared as previously described (Han et al., 2020) and stored at 4°C in the dark for further use.

### Preparation of the Colloidal Gold-Based Immunochromatographic Assay Strip

The colloidal GICA strip is composed of five parts (Yu et al., 2018), and its schematic diagram is shown in the Supplementary Data. Rabbit anti-goat IgG-coated colloidal gold probe was deposited onto a glass fiber pad at 3 μL/cm and dried at 37°C for 2 h to prepare the conjugate pad. The goat anti-rabbit IgG and purified rP20 were diluted with 50 mmol/L lead (pH 8.0) to the final concentration of 0.4 and 0.45 mg/mL, respectively. The goat anti-rabbit IgG was then dispensed onto the NC membrane with a volume of 1 μL/cm to form the control (C) line, and the test (T) line was formed using rP20 protein with the same procedure. Next, the NC membrane was blocked with 1% albumin from bovine serum and dried at 37°C for 2 h. The 100% pure cellulose fiber was used as the sample pad and absorbent pad. The sample pad, conjugate pad, NC membrane, and absorbent pad were overlapped and adhered to the polyvinyl chloride sheet to complete the fabrication of the strip. The assembled plate was then cut into 3-mm wide strip and sealed in a plastic bag.

When samples were added dropwise onto the sample pad and allowed to pass through the conjugate pad and NC membrane, the result could be visualized by the naked eyes in less than 10 min. If the samples contained the target analyte, the positive result showed two red lines, one next to C and one next to T. If the T region was colorless with a colored line appearing in the C region, it indicated that the result was negative. The absence of a red line in the C region was considered an invalid test.

### Specificity, Sensitivity, Repeatability, and Concordance Rate of the Test Strip

For the sensitivity test of the strip, Mccp standard-positive serum was diluted to 1:2, 1:4, 1:8, 1:16, 1:32, 1:64, and 1:128 with 0.01 mol/L PBS (pH 7.2). The same volume of standard-negative serum and standard-positive serum with different dilutions were added onto the sample pad to determine the sensitivity in detecting the serum samples with Mccp antibody. IHA was carried out with different dilutions to compare and evaluate the sensitivity of the test strip.

To evaluate the specificity of the GICA strip, Mo-positive serum, GTPV-positive serum, PPRV-positive serum, FMDV-A-positive serum, Mmc-positive serum, Mcc-positive serum, and *E. coli* BL21 (DE3)-positive serum were tested with the strip. One hundred microliters of each serum sample were added dropwise onto the sample pad and incubated for approximately 10 min at room temperature. Meanwhile, the samples were tested by IHA, and the results of the two methods were compared and analyzed.

For the repeatability analysis, 1:64 diluted standard-positive serum was detected with three batches of the GICA strips, and each batch was repeated 10 times.

For the concordance rate of the GICA strip test with the complement fixation test (CFT) and IHA test, a total of 106 clinical goat serum samples collected in the field from different goat farms were detected by the GICA strip, CFT, and IHA test. The procedure of the CFT and IHA test referred to the method described previously (Rahman et al., 2003; OIE, 2018). The results were analyzed to evaluate the accuracy of the test strip.

## Results and Discussion

DNA-based detection with high specificity and sensitivity has made the detection of Mccp much more reliable (Saeed and Osman, 2018), and the polymerase chain reaction (PCR) method has been proved to be a promising tool in diagnosing Mccp (Abraham et al., 2015). However, the traditional PCR method needs electrophoresis for post-analysis, during which there is a risk of contamination. The same drawback also exists in the loop-mediated isolated isothermal amplification method. Real-time PCR assay for the detection of Mccp overcomes aerosol contamination (Lorenzon et al., 2008), but it is not suitable for detecting Mccp in the field because of the dependence on specialized laboratory equipment and well-trained personnel. More recently, a recombinant polymerase amplification assay for rapid detection of Mccp was reported (Liljandera et al., 2015), which was highly specific and sensitive, and did not require prior DNA preparation, sophisticated equipment, or technical personnel. Hence, this method is feasible for the rapid diagnosis of CCPP in the field. In addition to the molecular methods, a number of studies on the serological detection methods of Mccp have been reported. The IHA test, with approval of the new veterinary drug registration certificate of the People’s Republic of China, is one of the earliest serological methods used to detect Mccp antibodies. However, the performance of this method was barely satisfactory because of the cross-reactivity among the organisms in the *M. mycoides* cluster (Samiullah, 2013). The CFT initially used in the diagnosis of CCPP by MacOwan and Minette (1976) was more specific but less sensitive than the IHA test (Samiullah, 2013). To improve the specificity and sensitivity, several enzyme-linked immunosorbent assay (ELISA) methods were developed to confirm the prevalence of CCPP (Peyraud et al., 2014; Jean de Dieu et al., 2019). Although with high specificity, sensitivity, and suitability for large-scale testing, such as the PCR method, the ELISA test relied on specialized laboratory equipment resulting in the unfeasibility for field diagnosis. However, the reliable sensitivity, specificity, field-level availability, and low cost are crucial for the diagnosis of CCPP, especially in undeveloped or developing countries (Yatoo et al., 2019).

**FIGURE 1:**
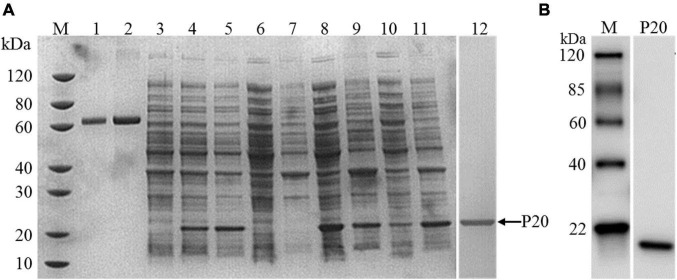
Expression, purification, and identification of rP20. **(A)** Sodium dodecyl sulfate–polyacrylamide gel electrophoresis analysis of expression of rP20. *M* Standard protein marker; *lane 1* albumin from bovine serum (1 μg); *lane 2* albumin from bovine serum (2 μg); *lane 3* lysates of non-induced recombinant bacteria cells; *lane 4* lysates of recombinant bacteria induced at 15°C for 16 h; *lane 5* lysates of recombinant bacteria induced at 37°C for 4 h; *lane 6* Supernatant of non-induced *E. coli*-Mccp-P20 lysate; *lane 7* Precipitation of non-induced *E. coli*-Mccp-P20 lysate; *lane 8* Supernatant of *E. coli*-Mccp-P20 lysate induced at 15°C for 16 h; *lane 9* Precipitation of *E. coli*-Mccp-P20 lysate induced at 15°C for 16 h; *lane 10* Supernatant of *E. coli*-Mccp-P20 lysate induced at 37°C for 4 h; *lane 11* Precipitation of *E. coli*-Mccp-P20 lysate induced at 37°C for 4 h. *lane 12* Purified rP20. **(B)** Western blot analysis of purified rP20 protein.

Herein, we developed a GICA strip for the rapid detection of Mccp antibodies present in the serum, which was independent of professional personnel and equipment and can be used for the diagnosis of CCPP onsite and in serological surveillance. The effectiveness of serum antibody detection methods depends largely on the quality of antigen used in the assay. Thus, preparations of antigen protein with good antigenic activity are essential for establishing reliable serological methods. The rP20 protein located on the outer membrane is the main specific antigen of Mccp and is different from the membrane protein of other organisms in the *M. mycoides* cluster. In this study, the rP20 protein was produced by *E. coli* expression system and expressed in soluble and inclusion body forms with a molecular weight of ∼20 kDa, which was confirmed by sodium dodecyl sulfate–polyacrylamide gel electrophoresis (Figure 1A), and the purified rP20 was verified by Western blot (Figure 1B). Moreover, a large scale of rP20 protein was expressed mainly in the soluble form at 15°C and existed as inclusion body form at 37°C with a shorter inducing time (Figure 1A). The Ni-NTA resins were used for the purification of His-tagged rP20 protein. However, the soluble rP20 was barely bound to the Ni-NTA resins and could not be purified by the HisTrap FF column successfully. In contrast, the denatured rP20 protein from inclusion bodies could be captured the Ni-NTA resins easily under the denaturing conditions, and the renatured recombinant protein also showed a high immunological reactivity.

**FIGURE 2:**
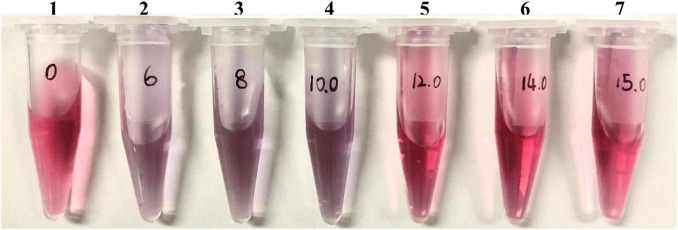
Optimal concentration of coating antibody. One to seven final concentrations of rabbit anti-goat IgG were 0, 6, 8, 10, 12, 14, and 15 μg/mL, respectively.

The reliability of the GICA results is affected by many factors, among which the quality of the colloidal gold particles, including uniformity and particle size, is one of the important parameters when preparing the GICA strip. The diameter of most colloidal gold particles varies from 5 to 150 nm, but for the application of gold conjugates in diagnostic assays, the diameter of gold particles usually ranges between 20 and 40 nm (Chaudhuri and Raychaudhuri, 2001). Smaller-sized colloidal gold particles are conducive to better mixing on the adsorption line, improving the detection sensitivity (Li et al., 2019). In this study, we prepared dispersed colloidal gold particles with a uniform diameter of 34 nm by using the ratio of 1:1.6 of hydrogen tetra-chloroaurate hydrate to trisodium citrate solution, which makes colloidal gold particles more stable and easier to flow on the membrane (Table 1). We also optimized the concentration of rabbit anti-goat antibody for colloidal gold labeling, to be 14 μg/ml, which is one of the important parameters affecting the color intensity of the T line and C line when the strip was used to detect serum samples (Figure 2).

**TABLE 1:** Diameter of colloidal gold particles produced under different reactants ratio.

Reactants ratio	1:0.5	1:1	1:1.6	1:2.5
Maximum absorption wavelength(λ)	537	532	529	521
Diameter of colloidal gold particles(nm)	53	39	34	16

**FIGURE 3:**
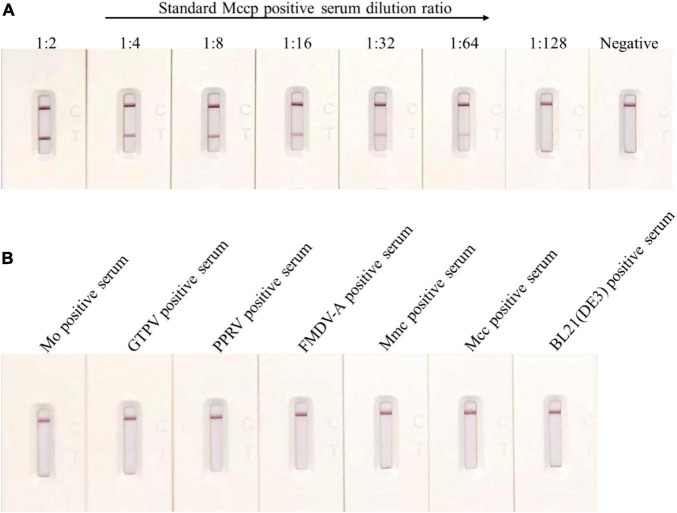
Sensitivity and specificity of the colloidal GICA strip. **(A)** The detection limit of the test strip was up to 1:64 dilutions of for the Mccp antibody positive serum samples. **(B)** The colloidal GICA strip did not cross-react with other pathogens positive serum.

The quality of the GICA strip was evaluated in this study, and sensitivity results demonstrated that this strip was still effective when the Mccp-positive serum samples were diluted 64 times (Figure 3A). Compared with the detection limit of up to 1:8 dilutions of the IHA test, the immunochromatographic strip had higher sensitivity (Table 2).

**TABLE 2:** Comparison of the sensitivity between the GICA strip and IHA test.

Dilution	GICA strip for Mccp antibodies	CCPP-IHA	
		Antibody titer*	Results
1:2	+	1:32	+
1:4	+	1:16	+
1:8	+	1:8	+
1:16	+	1:4	–
1:32	+	1:2	–
1:64	+	—	–
1:128	–	—	–
Mccp standard- negative serum	–	—	–

*Criteria of the CCPP-IHA test, if the titer was greater than or equal to 1:8; the serum sample was judged to be positive, and otherwise, it was judged to be negative.

The specificity of the test strip was analyzed by testing several common goat pathogens, including Mo-, GTPV-, PPRV-, FMDV- A-, Mmc-, Mcc-, and *E. coli* antibody-positive serum. The result revealed that the GICA strip had no cross-reaction with any positive serum of those pathogens (Figure 3B). In contrast, the CCPP-IHA test showed positive results in detecting the Mmc- and Mcc-positive serum samples (data not shown), which indicated that the GICA strip was more specific than the IHA method.

**FIGURE 4:**
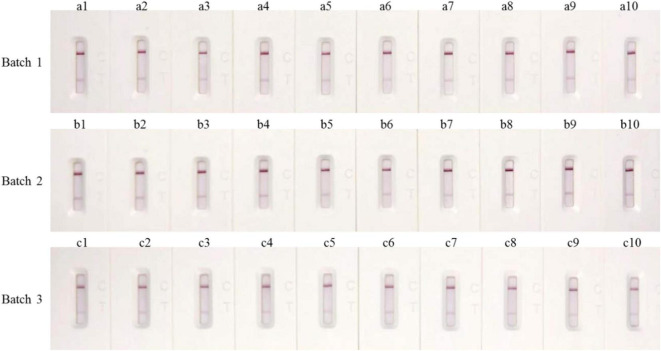
Repeatability of colloidal GICA strip.

To determine the repeatability of the GICA strip, 1:64 diluted Mccp standard-positive serum samples were used to test three batches of the test strip, and results showed that there were no significant differences among the three batches, and the C line or T line all displayed satisfactory color uniformity (Figure 4).

Although the CFT is the designated method for the diagnosis of CCPP in international trade and one of the diagnostic tests in endemic regions, its sensitivity and/or specificity for CCPP diagnosis is still disputed (Asmare et al., 2016; Yatoo et al., 2019). It is reported that the CFT failed in detecting 80–100% of animals experimentally infected with Mccp (March et al., 2000; Yatoo et al., 2019). In this study, both of the co-verification test results between the GICA strip and CFT or IHA by detecting 106 suspected clinical serum samples of Mccp showed a relatively low concordance rate. The positive rate was 53.77% by the GICA strip, 60.38% by the CFT, and 56.60% by the IHA test. The total concordance rate was 87.74% between the GICA strip and the CFT and 76.42% between the GICA strip and the IHA test (Table 3). Two reasons may be responsible for this result. First, the background of clinical serum samples is complex, including the inhomogeneous antibody level of Mccp in the samples and the existence of other pathogens of the *M. mycoides* cluster. Second, limitations of low sensitivity or specificity of the CFT and IHA test may lead to the possibility of cross-reaction with non-pathogenic *M. mycoides* cluster antibody or fail to detect Mccp antibody, which may be the main cause of low concordance rate among these methods. For further determination of the accuracy of this strip, it should be evaluated by the more sensitive and specific standard method such as ELISA.

**TABLE 3:** Detection of CCPP in clinical serum samples by GICA strip, CFT, and IHA test.

		CFT			CCPP-IHA		
		+	–	Total	+	–	Total
GICA strip	+	54	3	57	46	11	57
	–	10	39	49	14	35	49
Total		64	42	106	60	46	106
Concordance rate		87.74% = (54 + 39)/106			76.42% = (46 + 35)/106		

The GICA strip developed in this study was proved to be more sensitive and specific than the CCPP-IHA test, and it is convenient to use for the diagnosis of CCPP, although the sensitivity and specificity may be lower than the DNA-based detection methods and ELISA methods. Recently, the focus of CCPP diagnosis has been mainly on specificity, field applicability, and cost-effectiveness (Yatoo et al., 2019). The results indicated that the sensitivity, specificity, simplicity, and low cost of the GICA strip are sufficient for the rapid diagnosis of CCPP on site.

## Conclusion

In this study, a novel colloidal GICA strip was developed to rapidly detect Mccp antibodies in the field without relying on special equipment or skilled personnel, and it will be an economical and practical tool for the rapid and on-site diagnosis of CCPP in clinical samples.

## Data Availability Statement

The original contributions presented in the study are included in the article/supplementary material; further inquiries can be directed to the corresponding author/s.

## Ethics Statement

The animal study was reviewed and approved by the Animal Care and Use Committee of China Institute of Veterinary Drug Control.

## Author Contributions

ZZ, GQ, XC, and CW contributed to literature review and interpretation, manuscript writing, and design of the experiment. LW and JD contributed to protein expression and purification. QL provided the techniques for the strip development. ZS did the final approval of the manuscript. All authors contributed to the article and approved the submitted version.

## Conflict of Interest

The authors declare that the research was conducted in the absence of any commercial or financial relationships that could be construed as a potential conflict of interest.

## Publisher’s Note

All claims expressed in this article are solely those of the authors and do not necessarily represent those of their affiliated organizations, or those of the publisher, the editors and the reviewers. Any product that may be evaluated in this article, or claim that may be made by its manufacturer, is not guaranteed or endorsed by the publisher.

## Footnotes

1
https://www.oie.int/en/animal-health-in-the-world/oie-listed-diseases-2021/
